# Complete Genome Sequences of Six Isolates of the Oryctes rhinoceros Nudivirus

**DOI:** 10.1128/mra.00126-23

**Published:** 2023-07-05

**Authors:** Mitchell K. Weston, Charles A. Hefer, Jeanne M. E. Jacobs, Sean D. G. Marshall

**Affiliations:** a Resilient Agriculture, AgResearch Limited, Lincoln Research Centre, Christchurch, New Zealand; b Digital Agriculture, AgResearch Limited, Lincoln Research Centre, Christchurch, New Zealand; Katholieke Universiteit Leuven

## Abstract

Oryctes rhinoceros nudivirus, a double-stranded DNA virus of the family *Nudiviridae*, is an important biocontrol agent of the coconut rhinoceros beetle (Coleoptera: Scarabaeidae). We present the genome sequences of six isolates of Oryctes rhinoceros nudivirus collected from the Philippines, Papua New Guinea, and Tanzania between the years 1977 and 2016.

## ANNOUNCEMENT

We report the genome sequences of six isolates of Oryctes rhinoceros nudivirus (OrNV), a double-stranded DNA member of the family *Nudiviridae*, determined using the Oxford Nanopore Technologies (ONT; UK) platform. OrNV is used for biocontrol of the coconut rhinoceros beetle (CRB; Oryctes rhinoceros), an invasive pest of coconut and oil palm in Southeast Asia and the Pacific (1). Recent evidence suggests the emergence of a CRB genotype that is less susceptible to currently used OrNV isolates (2).

OrNV isolates (Table 1) were cultured and maintained in adherent cells of the Heteronychus arator DSIR-HA-1179 cell line (3) as previously described (2, 4). Infected cells were confirmed by microscopy, detached, collected by centrifugation, resuspended in water, and lysed by sonication, using 5 rounds of 20 s (MSE Sanyo Soniprep 150 sonicator; amplitude, 6.5 μm; 23 KHz). Lysate from isolates OrNV-PV505, -V23B, -X2B, and -PNG was treated with DNase I (Thermo Fisher Scientific, New Zealand) to remove host cell DNA. OrNV-S2A and -DUG42 were not treated with DNase I. Lysates were treated with RNase A and proteinase K (New England Biolabs, New Zealand), and the DNA was purified as previously described (5).

**TABLE 1:** Sequencing results of six OrNV isolates

OrNV isolate	Isolation location	Yr isolated	GenBank accession no.	SRA accession no.	Genome size (bp)	No. of predicted genes	No. of high-quality reads	Avg coverage (×)
OrNV-PV505	Philippines	1977	OP831188	SRR21977632	125,879	115	3,272	31.7
OrNV-V23B	Philippines	1980	OP831190	SRR21977630	123,988	114	3,163	30.7
OrNV-X2B	Philippines	1983	OP831191	SRR21977629	125,660	118	2,994	27.9
OrNV-PNG	Papua New Guinea	1988	OP831187	SRR21977633	124,855	116	3,503	34.0
OrNV-S2A	Tanzania	1981	OP831189	SRR21977631	125,830	140	3,524	41.3
OrNV-DUG42	Philippines	2016	OP831186	SRR21977634	125,840	114	3,496	41.3

Sequencing libraries for OrNV-PV505, -V23B, -X2B, and -PNG were prepared using genomic DNA by ligation sequencing with a PCR barcoding protocol (SQK-LSK110 and EXP-PBC001, respectively; ONT). Libraries for OrNV-S2A and -DUG42 were prepared using the genomic DNA by ligation sequencing protocol (SQK-LSK110; ONT). All libraries were prepared without DNA shearing. Sequencing was performed on a MinION MK1B instrument using FLO-MIN106 (R9.4.1) flow cells. OrNV-S2A was sequenced using the MinKNOW v21.05.25 adaptive sequencing function to enrich for virus reads by alignment to the OrNV genome (6) (GenBank accession number MW298154). Three flow cells were used, one for the combined barcoded library (OrNV-PV505, -V23B, -X2B, and -PNG) and one each for OrNV-S2A and -DUG42, generating 6.70 million reads (*N*_50_, 1,750 bp), 1.96 million reads (*N*_50_, 692 bp), and 5.15 million reads (*N*_50_, 2,150 bp), respectively. Base calling was performed using Guppy v4.3.4 (7) for OrNV-PV505, -V23B, -X2B, and -PNG and Guppy v5.0.14 for OrNV-S2A and -DUG42. Data were subjected to quality control using pycoQC v2.5.2 (8).

Viral DNA sequences were computationally identified for OrNV-DUG42 by aligning the sequenced library to the OrNV genome (6) (GenBank accession number MW298154) using Magic-BLAST v1.6.0 (9) before processing. Using NanoFilt v2.8.0 (10), 100 bp was trimmed from each end of the reads, and the high-quality reads (score, >12) were filtered for chimeric reads using Yacrd v1.0.0 (11) before error correction using Canu v2.2 (12). Reads longer than 250 bp were assembled using Flye v2.9 (13) with 10 iterations of polishing, circularized using Circulator v1.5.5 (14), and polished using Pilon v1.24 (15). The genomes were annotated using Prokka v1.14.6 (16) with the protein sequences found under GenBank accession numbers EU747721, MW298153, MT150137, MZ727584, AH015832, and MN623374.

The six genomes were each assembled into a single contig with 41.6% G+C content. The isolate information, sequencing metrics, and accession numbers are listed in Table 1. The genome lengths ranged from 123,988 bp to 125,879 bp, with the average coverage ranging from 27.9× to 41.3×.

### Data availability.

The complete genome sequences have been deposited at GenBank under accession numbers OP831186, OP831187, OP831188, OP831189, OP831190, and OP831191. The raw reads were deposited in the SRA under accession numbers SRR21977629, SRR21977630, SRR21977631, SRR21977632, SRR21977633, and SRR21977634.
